# Private capital in a public field: a qualitative study of philanthropic influence in Germany’s global health governance

**DOI:** 10.3389/fpubh.2026.1960171

**Published:** 2026-09-21

**Authors:** Henrike Gerdkamp, Hanna Luetke Lanfer, Victoria Saint

**Affiliations:** School of Public Health, Bielefeld University, Bielefeld, Germany

**Keywords:** agenda-setting, gates foundation, Germany, global health governance, philanthropy, power, qualitative research, Wellcome Trust

## Abstract

**Background:**

International philanthropic foundations have become prominent actors in global health, influencing funding priorities, institutional partnerships, and governance practices at a scale that increasingly rivals many state actors. While their role in multilateral organisations and low- and middle-income countries (LMICs) has been widely debated, less is known about how these actors operate within high-income donor countries themselves. Over the past two decades, Germany has emerged as a key contributor to global health initiatives and has developed close working relationships with international foundations, particularly the Gates Foundation and the Wellcome Trust. This study examines how global health stakeholders in Germany perceive the evolving role and influence of international philanthropic foundations within the country’s domestic global health landscape and explores the implications for global health governance.

**Results:**

Across political, academic, civil society, private sector, and philanthropic stakeholders, four principles roles were attributed to international philanthropic foundations: strategic funding, thematic prioritisation, knowledge brokerage, and convening. Foundations were widely perceived as simultaneously enabling and constraining, providing resources, political momentum and continuity in global health engagement, particularly during periods of political uncertainty, while also concentrating agenda-setting power and shaping the boundaries of legitimate intervention. Five principal domains of perceived impact were identified: priority- and agenda-setting, governance fragmentation, organisational dependency, stabilisation of global health, and legitimacy debates. Influence was perceived to operate not only through financial resources but also through networks, expertise, and symbolic credibility, with growing dependency on private foundations described as deepening structural asymmetries within the field.

**Conclusion:**

The German case illustrates how international philanthropic foundations interact with public development institutions within a major donor state, simultaneously strengthening and reshaping national engagement in global health. The dynamics identified, including strategic alignment, thematic narrowing and organisational dependency, are not confined to LMIC contexts but are structurally embedded within the broader architecture of global health governance, reinforcing critique of the donor/recipient binary that often frames this field of inquiry. As public financing contracts and philanthropic actors assume a more prominent role in filling resulting gaps, greater transparency, safeguards for pluralism, and governance frameworks that preserve accountability and equity in global health become increasingly important.

## Introduction

### Philanthropy and global health governance

Over the past two decades, large international philanthropic foundations have become central actors in global health governance. Institutions such as the Gates Foundation (formerly Bill and Melinda Gates Foundation) and the Wellcome Trust shape funding priorities, strategic policy and research agendas, and multilateral partnerships at a scale that rivals many state actors (1–3). Their rise coincides with broader transformations in development assistance, including the proliferation of public–private partnerships, increasing reliance on earmarked funding, and the growing complexity, and at times fragmentation, of global health financing architectures (4–6).

In global health and development assistance governance, philanthropic foundations occupy a hybrid position. They are neither state agencies nor traditional civil society organisations, yet they deploy substantial financial resources and maintain close relationships with multilateral institutions and partnerships such as the World Health Organization (WHO), Gavi, the Global Fund and the Coalition for Epidemic Preparedness Innovations (CEPI), where they have become firmly embedded decision-making structures (7, 8). Analyses of funding flows have shown that private philanthropies account for significant shares of development assistance for health in selected sectors, while also influencing institutional priorities and policy design through market-oriented and managerial logics—sometimes termed *philanthrocapitalism*—that privilege measurable impact, scalability and technological innovation, over structural reforms and health system strengthening (9–11). In a global governance system where donor interests already shape aid allocations, the growing authority of private foundations further complicates questions of democratic accountability, legitimacy and public oversight (12–15).

These developments have gained increasing urgency in the context of recent geopolitical and fiscal shifts affecting global health and development assistance. Reductions in official development assistance by several high-income countries, including significant funding contractions in the United States, the United Kingdom, France and Germany, alongside periods of withdrawal or disengagement from multilateral platforms—such as the withdrawal of the United States from the WHO and the institutional realignments associated with Brexit—have intensified financial uncertainty and institutional volatility within the global health system (16, 17).

While extensive research has analysed philanthropic influence on multilateral institutions and low- and middle-income countries (LMICs) (18, 19), comparatively little attention has been paid to how foundations operate within high-income donor states themselves. Recent analyses have examined both philanthropic funding priorities in specific sectors and the strategic role of foundations in shaping research partnerships, concluding that further actor-centred investigation is needed to understand how philanthropic agenda-setting and operational approaches influence governance outcomes (9, 20). Work on philanthropic ‘network diplomacy’ in European donor countries has further demonstrated how the Gates Foundation builds strategic relationships with governments and positions itself within national policy networks (21); yet less is known about how philanthropic actors are perceived within domestic policy communities and how their presence reshapes field-level dynamics inside donor states. Systematic, actor-centred analyses of these internal dynamics remain limited. In particular, it remains unclear how philanthropic foundations are perceived by actors across different sectors within high-income donor states, what roles they are understood to perform within domestic global health governance, and through which pathways these roles may translate into perceived impacts.

### Germany as a donor-state case

Germany provides a particularly relevant case for examining how philanthropic capital interacts with public development institutions. Until the early 2000s, Germany played a relatively limited role in international health policy (22). Over the past 15 years, however, it has emerged as one of the largest contributors to multilateral health initiatives, significantly expanding its political and financial engagement, alongside a growing and increasingly diverse domestic global health landscape (23, 24). Global health gained prominence during Germany’s G7 and G20 presidencies and through increased voluntary contributions to the World Health Organization, alongside substantial support for Gavi and the Global Fund (25, 26). The COVID-19 pandemic further accelerated this trajectory by underscoring the transnational nature of health threats and the centrality of multilateral coordination. During this period, Germany was widely perceived as a stabilising multilateral actor, particularly in the context of funding volatility and the temporary withdrawal of the United States from the WHO during the first Trump administration, reinforcing its position as a key supporter of multilateral health governance (22, 23, 27). Germany’s evolving role in global health—from a comparatively cautious actor to a prominent multilateral donor and policy shaper—has been systematically documented in key analyses tracing its institutional, financial, and strategic milestones from the early 2000s through 2023 (22, 23, 28).

Berlin has evolved into a hub for transnational health diplomacy and policy exchange, hosting initiatives such as the World Health Summit, the WHO Hub for Pandemic and Epidemic Intelligence, and the Global Antimicrobial Resistance Research and Development Hub (Global AMR R&D Hub) (29–31). In parallel, international philanthropic foundations have institutionalised their presence in the country. Since the mid-2010s, both the Gates Foundation and the Wellcome Trust have become increasingly active in and with Germany, establishing offices in Berlin in 2018 and 2019 respectively, and developing structured relationships with governmental, academic, and civil society actors (21). Publicly available grant records (retrieved in August 2026) show that the Gates Foundation’s Committed Grants database lists 256 grants to recipients in Germany, and Wellcome’s grants data list 58 grants to German recipients between 2005 and 2026 (32, 33). Grant activity to Germany has grown accordingly since the mid-2010s: the number of Gates Foundation grants to German recipients more than tripled, from 55 grants between 2005 and 2014 to 201 between 2015 and 2026, while Wellcome’s grant volume to German recipients rose from £4.6 million to £75.4 million across the same two periods. At the same time, the growing prominence of Germany’s pharmaceutical, biotechnology and other private sector stakeholders within global health partnerships have generated debate about potential tensions between national industrial interests, intellectual property regimes, and commitments to global health equity (22, 34–36).

Germany offers a particularly revealing setting for examining how philanthropic actors are embedded in Germany’s global health landscape and how they interact with stakeholders from other sectors, including government, academia, civil society, and the private sector. Against this background, attention is given to both the roles attributed to international philanthropic organisations and the impacts that stakeholders associate with these roles. Roles here refer to the functions or positions that stakeholders attribute to philanthropic organisations within Germany’s global health landscape. Impacts refer to the effects or influence associated with the activities of philanthropic organisations according to multisectoral stakeholders. For instance, performing strategic funding of specific topics (role) may become visible in the agendas of NGOs involved in global health (impact). Hence, we ask:

RQ1: What roles do stakeholders attribute to international philanthropic organisations in Germany’s global health landscape?

RQ2: What impacts do stakeholders perceive international philanthropic organisations have on Germany’s global health landscape?

## Methods

### Study design

This study employed a qualitative interview design to explore the perceived role and influence of international philanthropic foundations in Germany’s global health field over the past 15 years.

### Sampling and recruitment

This exploratory study applied purposive sampling to recruit individuals with in-depth knowledge of or direct involvement in global health policy and governance in Germany. Sample size was guided by the principle of information power as described by Malterud and colleagues (37). Given the study’s analytical aims, the specificity of an expert sample in which each participant contributed first-hand knowledge of the field was the guiding principle of our recruitment approach. Participants were selected across five actor sectors: government institutions, academia and research, civil society organisations (CSO), private sector and international philanthropic organisations (with offices in Germany). Initial contact was established via professional networks, publicly available affiliations, and institutional directories. Snowball sampling was used to identify further relevant actors. Inclusion criteria were longstanding professional experience in the field of global health and familiarity with the presence and activities of international philanthropic foundations in Germany. Recruitment was concluded when all pre-specified actor domains were represented and the interviews did not yield substantively new aspects to the existing material.

In total, 11 expert interviews were conducted with participants holding senior or mid-level positions across all five actor domains. Each sector was represented by at least two participants, ensuring a balanced inclusion of diverse institutional perspectives and capturing a broad range of positions and insights relevant to the evolving role of philanthropic actors in Germany’s global health field. To protect participant anonymity, gender and other demographic variables were not explicitly collected. Recruitment outcomes varied across sectors (see Table 1). Non-participation was shaped in part by the sensitivity of the topic within a small and closely connected professional field as several actors, especially from government and philantrophic organisations, declined out of concern that their views could be attributed to them despite pseudonymisation, or anticipated professional disadvantage from taking part.

**Table 1:** Overview of participants from different sectors (*N* = 11).

Actor domains	Contacted (n)	Participated (n)
Private sector	2	2
Civil society organisations (CSO)	4	2
Government institutions	9	2
Academia/research	5	3
International philanthropic organisations	9	2

### Data collection

Interviews were conducted between June and July 2025 via video conference in English. Informed consent was obtained in writing prior to participation. Interviews followed a semi-structured guide with open-ended questions on participants’ experiences with international philanthropic actors, perceived influence on the German global health agenda, and changes in actor constellations over time. Questions also included reflections on legitimacy, governance, and the interplay between public and private authority. The average interview duration was 45 min, ranging from 25 to 60 min. All interviews were audio recorded, transcribed verbatim, assisted by AI (f4x) and checked manually for accuracy. During the transcription, all interviews were pseudonymized.

### Data analysis

Data were analysed using qualitative content analysis by Kuckartz supported by the software f4analyse (38). The coding framework was structured around the two research questions. Subcategories were developed deductively from the research questions, interview guide and relevant concepts from the literature on philanthropic influence. It was subsequently refined inductively based on the interview material. The first author conducted the initial coding and the coding frame was refined in two collaborative rounds with the co-authors, including joint discussions of selected transcript segments to ensure clarity, consistency, and conceptual alignment. After finalising the coding frame (see Table 2), the first author coded the material twice at two different points in time.

**Table 2:** Coding frame.

Code	Definition
RQ1: Roles attributed to international philanthropic organisations in Germany’s global health landscape	
Strategic funding	Foundations perceived as grant-makers; funding described as selective and strategic, broadly dispersed to generate dense networks aligned with philanthropic priorities
Thematic prioritisation	Foundations associated with a concentration around specific disease areas and technical interventions, reinforced through outcome-driven monitoring and evaluation practices; structural and health systems approaches perceived as comparatively marginalised
Knowledge brokerage	Foundations perceived as shaping knowledge (epistemic) and policy environments through research commissioning, promotion of metrics and analytical frameworks, and narrative framing; described as operating indirectly via universities, NGOs and governments rather than through direct knowledge production
Convening	Foundations described as facilitators of high-level cross-sector exchange through formal platforms and informal bilateral diplomacy; personal reputations of individual figures (e.g., Bill Gates, Jeremy Farrar) identified as amplifying convening capacity and political access
Change over time	Perceived trajectory of increasing institutionalisation of philanthropic foundations within Germany’s global health landscape over the past fifteen years; described as mutually reinforcing, with foundations gaining proximity to decision-makers while German actors benefited from additional expertise and networks
RQ2: Impacts international philanthropic organisations are perceived to have on Germany’s global health landscape	
Priority- and agenda-setting	Perceived impact of philanthropic funding on sustaining and narrowing the thematic focus of Germany’s global health engagement; associated with homogenisation of global health discourses and reorientation toward interventions aligned with philanthropic logics of measurability, scalability and demonstrable impact
Governance fragmentation	Perceived as contributing to the proliferation of parallel, disease-specific initiatives alongside multilateral institutions, generating coordination challenges and a redistribution of institutional authority away from organisations with broader, democratic mandates
Organisational dependency	Perceived reshaping of power relations through strategic alignment with philanthropic funding priorities; described as structurally embedded in funding realities, with organisations adjusting their thematic focus in response to financial incentives
Stabilisation of global health	Perceived as providing continuity and financial leverage in a context of shrinking public budgets; recognised as catalytic actors in replenishment processes and gap-fillers preventing programme collapse, while simultaneously deepening structural dependencies
Legitimacy debates	Perceived influence on democratic accountability and public trust, including systemic critique of framing philanthropic prominence as a consequence of insufficient public financing and wealth concentration within the hands of few wealthy individuals

## Results

### Roles attributed to philanthropic foundations in Germany’s global health landscape (RQ1)

Four principal roles attributed to philanthropic foundations emerged from the analysis: *strategic funding, thematic prioritisation, convening, and knowledge brokerage*. Perceptions of these roles were dynamic, with participants noting shifts in foundation influence and visibility over the past decade, captured under a final code *change over time*.

*Strategic funding*: Across all actor domains, international philanthropic foundations were first and foremost described as grant-makers. Funding relationships structured interactions, visibility and proximity within the field. As one representative of an international philanthropic foundation put it:

“I think it’s mostly the funding side. If we don’t have a funding relationship we don’t have much of an interaction with people.” (P2, Philanthropic sector).

However, funding was not portrayed as neutral resource allocation. Participants repeatedly framed it as strategic and selective, with grants described as deliberately distributed across advocacy organisations, research institutions, UN agencies, think tanks and even for-profit companies. This broad dispersion was emphasised by several interviewees as generating dense advocacy and policy networks that were indirectly shaped by philanthropic priorities. Some actors characterised these funding constellations as forming an “self-sustaining industry.”

“It’s sometimes almost a circular industry now, where Gates [Foundation] puts money into the Global Fund, the Global Fund puts money into NGOs, and those NGOs then are advocating for money from a government. … It’s kind of feeding itself. It’s a self-sustaining industry.” (P6, Government sector).

At the same time, international philanthropic foundations’ financial power was described as symbolically overpowering. One participant recalled a replenishment conference in Berlin where a 50€ million pledge by the German Chancellor was followed by a USD 1.5 billion announcement by Bill Gates, calling it “a shaming moment for the Government” (P1, CSO sector). Such moments were interpreted as signalling not only financial scale but hierarchical positioning within global health diplomacy.

*Thematic prioritisation*: Beyond funding volumes, participants attributed a strong thematic priorisation to international philanthropic engagement in Germany’s global health landscape. The Gates Foundation in particular was consistently associated with a focused portfolio of infectious diseases, especially “HIV/AIDS, tuberculosis, malaria and polio” (P2, Philanthropic sector). Several interviewees described this portfolio as shaping not only funding streams but also the broader architecture of political mobilisation and advocacy in Germany.

The Wellcome Trust, by contrast, was portrayed as historically rooted in biomedical research funding, though increasingly expanding its scope. One respondent characterised the Wellcome Trust as having traditionally adopted a predominantly clinical and biomedical approach, while noting a recent shift towards greater engagement with global health governance and upstream policy issues, including climate change (P11, Academic sector). Despite this diversification, both foundations were described as maintaining a strong commitment to scientific and technical interventions.

Several respondents, particularly from civil society and academic sectors, criticised international philanthropic funding for prioritising biomedical innovation, measurable outputs and eradication strategies at the expense of approaches addressing social determinants of health, rights-based frameworks or health system strengthening.

“What we criticised a lot is that the strict focus on, like, technical solution issues of the Gates Foundation—to a certain extent also Wellcome because Wellcome is much more a scientific funding organisation, so this strong belief that you need the right technical solutions for health problems that we would say rather has a social dimension.” (P5, CSO Sector).

Monitoring and evaluation practices were cited as emblematic of, and reinforcing, this prioritisation. Foundations were described as strongly outcome-driven, institutionalising target-setting and quantification as central governance tools. As one interviewee put it succinctly, “He really wants outcomes, Gates” (P6, Government sector). While some interviewees interpreted this results-orientation as contributing to professionalisation and accountability, others viewed it as reinforcing a narrowing of what counts as legitimate intervention, with consequences for the broader field (taken up under RQ2). Structural reforms and systemic approaches were described as comparatively disadvantaged because their impact is harder to demonstrate, potentially reducing willingness to invest in them.

*Knowledge brokerage*: Participants also attributed an important epistemic role to international philanthropic foundations, including commissioning research, funding universities and think tanks, and supporting institutions such as the Institute for Health Metrics and Evaluation. Through these investments, international foundations were described as shaping discourse, priority-setting language and dominant policy frames:

“The Gates Foundation has been much more influential in terms of shaping the stories we tell in global health, in terms of shaping the narratives, in terms of shaping the discourse, the language about global health and how we set priorities and what interventions we choose to prioritise and deprioritise.” (P11, Academic sector).

This influence was described as operating through several mechanisms. These included the commissioning and funding of research programmes, the development and promotion of widely used metrics and analytical frameworks, and the dissemination of findings through academic, policy and media channels. Through these processes, foundations were seen as shaping which problems become visible, how they are framed, and which interventions are considered legitimate policy responses. Critically, this was not portrayed as direct knowledge production. Rather, international philanthropic foundations were seen as leveraging external expertise and operating through universities, NGOs, media and governments, selectively amplifying forms of evidence and policy narratives that align with their strategic priorities.

Several participants further referred to international philanthropic foundations’ influence over intellectual property regimes during the COVID-19 pandemic, arguing that these foundations and the Gates Foundation in particular defended existing intellectual property structures, and thereby shaped the boundaries of reform debates.

*Convening*: Another recurring role concerned international philanthropic foundations as conveners and facilitators of elite exchange. Interviewees described how foundations host conferences, create platforms for cross-sector dialogue and bring together representatives from government, academia, civil society and the private sector. These spaces were portrayed as strategically curated to enable agenda alignment and coalition-building.

Yet convening was not limited to formal settings. Several participants emphasised that it often operated “behind closed doors” (P7, Academic sector), through bilateral meetings and informal diplomacy. Such interactions were described as particularly consequential for shaping political priorities and building trust-based relationships with decision-makers.

A key enabler of this convening function was the symbolic weight carried by individual figures associated with foundations. Bill Gates for the Gates Foundation and Jeremy Farrar for the Wellcome Trust were mentioned as symbolic reference points: Gates’ presence in particular was portrayed as capable of attracting political leaders and media attention, thereby strengthening the visibility and agenda-setting capacity of international philanthropic foundations across the field:

“If you want to announce something as a politician you need publicity, ideally international publicity. Bill Gates and his foundation provide that in this situation.” (P3, Private sector).

“Jeremy Farrar is a very credible figure and he steered the Wellcome Trust for some time and I think that the Wellcome Trust has also benefited from his reputation.” (P6, Government sector).

These accounts suggest that the convening role of international philanthropic foundations operates through relational and symbolic forms of power—extending well beyond funding or knowledge production into the informal architecture of elite networks.

*Change over time*: Across interviews, the past 15 years were described as a period of increasing institutionalisation of international philanthropic foundations within Germany’s global health landscape. Several participants pointed to the establishment of German offices by both the Gates Foundation and the Wellcome Trust, particularly in the context of Brexit, and as signalling Germany’s growing relevance as a central actor in global health diplomacy.

“Gates was still here increasing its activities, the Wellcome Trust started increasing its role and of course with Brexit both really exploded, so five years ago or so.” (P1, CSO sector).

This expansion was not framed as a unilateral imposition. Rather, one international philanthropic representative suggested that foundations’ increasing presence in Berlin also responded to demands from German governmental actors seeking closer collaboration and policy exchange. In this sense, institutionalisation was portrayed as mutually reinforcing: foundations gained proximity to political decision-makers, while German ministries benefited from access to additional expertise, networks and financial leverage.

“In the Gates Foundation’s history, more offices have been opened in the past five years than in the preceding ten years and it’s mostly that the partner government specifically asks for it. They say we find it difficult to kind of see [Gates] as a reliable and trusted partner if[they’re] in a time zone that is nine hours away from us.” (P2, Philanthropic sector).

Looking ahead, the announced closure of the Gates Foundation in 2045 was mentioned by some participants as a potential future turning point. While its long-term implications were described as uncertain, interviewees reflected on what such a withdrawal might mean for organisations and initiatives that have become structurally aligned with philanthropic funding. In particular, participants questioned whether existing programmes, partnerships and advocacy structures could be sustained, and whether other public or private actors would be able or willing to fill the resulting financial and institutional gaps. Some went further, raising concerns about who might emerge to occupy this space, noting that the departure of such a prominent actor could open the field to new philanthropic actors whose values, priorities and approaches to global health governance may differ substantially from those currently shaping the field.

### Perceived impacts of philanthropic foundations on Germany’s global health landscape (RQ2)

Analysis identified five principal themes of perceived impact by international philanthropic foundations: priority- and agenda-setting, governance fragmentation, organisational dependency, stabilisation of global health and legitimacy debates.

*Priority- and agenda-setting*: A central perceived impact concerned the reinforcement of specific priorities in Germany’s global health engagement. Interviewees across domains described international philanthropic funding as contributing to sustained political and organisational attention to a comparatively narrow set of issues. While some participants framed this as stabilising commitments and mobilising resources, others described it as narrowing the thematic bandwidth of German global health debates and programmes.

“I don’t think the German government would have been as committed if the Gates Foundation and others wouldn’t have constantly said, ‘guys, we’ve been doing this for a long time, let’s do it for another year and another year.’” (P2, Philanthropic sector).

“There’s been a kind of homogenisation of thinking, narratives, discussion debates in global health and it’s certainly crowded out the voices of many other actors with different perspectives.” (P11, Academic sector).

Participants described these dynamics as reshaping how priorities are translated into political commitments and organisational strategies. In particular, an emphasis on measurable outcomes and technical interventions was seen as influencing which types of programmes gain traction within Germany’s global health engagement. This results-oriented logic was associated with disease-specific, vertically organised programmes and eradication strategies, while structural and health systems approaches were perceived as receiving comparatively less attention within policy debates and funding landscapes. Several interviewees suggested that this dynamic disadvantages long-term, politically complex reforms that require institutional change, redistribution of resources or engagement with the social determinants of health.

“So, this global health free market really benefited while groups that are working on the determinants of health, rights-based approaches, equity, … and health workforce development, health system strengthening, universal health coverage they have been pushed to the sides it’s a very selective technocratic approach of global health.” (P8, Academic sector).

Several participants described these dynamics as shaping not only funding patterns but the broader structure of political feasibility within the German global health landscape. Interventions associated with measurable targets, technological solutions and clear attribution of impact were described as easier to mobilise support for, while systemic reforms requiring institutional change, redistribution of resources or engagement with social determinants were perceived as more difficult to advance. Cumulatively, these effects were described as contributing to a gradual reorientation of Germany’s global health agenda towards interventions that align with philanthropic logics of measurability, scalability and demonstrable impact.

*Governance fragmentation*: Participants also attributed governance-level impacts to international philanthropic expansion. A recurring theme concerned the proliferation of disease-specific initiatives and partnership-based structures, in which Germany engages and that operate alongside established multilateral institutions. This development was described as contributing to fragmentation. One interviewee referred to the “problem of establishing parallel systems” (P5, CSO sector) where projects differ in accounting and reporting structures, generating coordination problems across the field.

Several respondents suggested that philanthropic expansion has also altered institutional authority within global health governance. Disease-specific funding streams were seen as shifting influence away from multilateral organisations with broader mandates toward more narrowly focused partnerships. As one government-sector participant reflected:

“So Gates, in a way, pumps money into certain disease specific approaches. …the same time, it’s led to a fragmentation of the global health landscape, especially the global health financing landscape and the weakening of WHO.” (P6 Government sector).

In this account, philanthropic funding simultaneously enables progress in targeted areas while redistributing authority within the global system—particularly vis-à-vis WHO. Several interviewees described this shift not as an intentional displacement, but as a structural consequence of earmarked, partnership-based financing models that operate outside core multilateral budgets.

These governance effects were not portrayed as uniformly negative. Participants acknowledged that parallel structures have mobilised substantial resources, accelerated innovation and increased political visibility for certain diseases, with some initiatives described as more agile and results-oriented than traditional multilateral mechanisms. The overall picture was therefore one of structural ambivalence: enabling scale and technical progress in priority domains, while complicating coordination, increasing fragmentation and challenging the coherence of multilateral governance arrangements.

*Organisational dependency*: International philanthropic presence was widely perceived as altering power relations within Germany’s global health landscape. Several participants described strategic alignment dynamics, where organisations adjust priorities in response to funding incentives. As one interviewee explained, “you are following the money, you are following the funders priority” (P3, Private sector). Another framed this more broadly as organisations having “danced to the tune that the Gated Foundation has played” (P11, Academic sector).

Such adaptation was not necessarily portrayed as coercive but as structurally embedded in funding realities. In a context of limited alternative resources, international philanthropic priorities were seen as shaping what is politically and programmatically feasible—a dependence captured starkly by one participant:

“If you have the choice between confronting a team of 20 people in your organisation’s office with the prospect of ‘we can’t go on with money from Gates, they then lose their jobs and may have to move cities’ or you have the chance to take the Gates money—what will you take? You take the money and you swallow critique, but that is not exclusive to Gates; it’s a sign of a dependency of a sector.” (P3, Private sector).

At the same time, international philanthropic foundations were credited with enabling new forms of cross-sector cooperation. Their convening role was repeatedly emphasised: as one participant reflected, international philanthropic actors “allowed actors across sectors in Germany to speak together” (P8, Academic Sector), facilitating dialogue between government, civil society, research institutions and private actors.

Several interviewees also noted a broader normative shift, with international philanthropic funding described as contributing to a normalisation of participation of non-state actors in public policy arenas. In this sense, international philanthropic foundations were perceived as actors reshaping norms of participation and legitimacy within global health governance.

“Philanthropic funding is facilitating an acceptance of non-state actors in public affairs.” (P8 Academic sector).

*Stabilisation of global health*: Participants situated international philanthropic influence within a broader context of shrinking public budgets, declining official development assistance (ODA) commitments and increasing fiscal uncertainty. In this environment, international philanthropic foundations were described as occupying a structurally ambivalent position: simultaneously stabilising global health financing while reinforcing new dependencies within the system.

Several interviewees emphasised the catalytic effect of large international philanthropic pledges, as the financial scale of the Gates Foundation could actively shape governmental commitments, including in Germany:

“I mean, also because the Gates Foundation is able to do this with so much money that basically it forces national governments to give more than what the Gates Foundation pledges.” (P4, Private sector).

In replenishment processes in particular, this dynamic was described as structuring political expectations and influencing the scale of public contribution. Growing reliance on international philanthropic resources was viewed as a direct consequence of contracting public budgets. Several participants referred to funding gaps resulting from German and international budget cuts, anticipating that “more organisations will turn to philanthropy to fund the gap” (P8 Academic sector). In such contexts, foundations were seen as providing continuity and preventing abrupt programme collapse.

Yet this stabilising role was closely tied to concerns about concentration and dependency. Beyond a certain funding threshold from a single actor, organisational autonomy was perceived as increasingly constrained:

“If you claim to be an independent organisation, but your funding is over 50% from one organisation, in a sense you’re forced to become some kind of sock puppet for the interests of the funding organisation.” (P4, Private sector).

This dependency was not framed primarily as overt pressure, but as structural alignment driven by survival imperatives—subtly reshaping the boundaries of permissible dissent within the field. Importantly, philanthropic representatives themselves acknowledged the limits of substitution:

“Philanthropy can’t replace government funding, it’s just too much.” (P9, Philanthropic sector).

Despite their scale, foundations were widely perceived as incapable of compensating for sustained reductions in public multilateral financing. Philanthropic foundations thus emerged from these accounts as gap-fillers in times of fiscal constraint, yet not as systemic substitutes for public authority. Their stabilising function was recognised and, in some cases, valued. However, this very stabilisation was described as deepening asymmetries in agenda-setting power and reinforcing structural dependencies within Germany’s global health landscape. Philanthropic influence thus appeared not merely additive, but transformative in shaping the political economy of priority-setting under conditions of financial scarcity.

*Legitimacy debates*: Finally, participants reflected on the implications of international philanthropic influence for democratic legitimacy and public trust. Foundations were repeatedly characterised as powerful yet unelected actors capable of shaping national and global priorities. The symbolic authority described under convening was seen as compounding this legitimacy question, as influence derived from reputation and access rather than democratic mandate.

“Of course, they are not democratically elected or legitimised and I think they have done rather good stuff, but they also have certain agendas and because they have so much money, more than nation states, they can actually influence their agenda.” (P6, Government sector).

Several respondents framed this dynamic within a broader systemic critique that shifted the focus away from individual foundations and toward the political economy of global health financing. Rather than positioning philanthropic influence primarily as a problem of private power, some interviewees argued that it reflects insufficient public financing of multilateral institutions—with philanthropic prominence interpreted as a consequence of state retreat or underfunding, rather than the root cause of governance imbalance.

Others extended this critique further, describing philanthropy as embedded in deeper structural inequalities. One interviewee characterised foundations as “a symptom of a global problem,” arguing that extreme wealth concentration and tax avoidance create the very conditions in which private charity becomes necessary: billionaires “should probably pay their taxes and then governments could also spend more on health and global health” (P6, Government sector). The most pointed formulation came from a civil society participant who questioned the broader model of mobilising private wealth for public good:

“You need to mobilise private money for solving public problems rather than giving the money to the public sector through taxation, for example. And this is the question we would ask—not so much is Gates doing the right thing, but why do we need a Gates Foundation?” (P5, CSO sector).

These accounts converge as a picture of philanthropic impact in Germany’s global health landscape as structurally transformative yet normatively contested: simultaneously enabling political commitment and generating debate over power, accountability and the boundaries of private authority in public health governance.

## Discussion

This study examined how stakeholders across Germany’s global health landscape perceive the role and impact of international philanthropic foundations. We conducted semi-structured expert interviews with professionals across five actor domains: government, academia, civil society, the private sector, and international philanthropic organisations. The findings reveal that philanthropic foundations are seen as strategically important institutions for Germany’s global health engagement across sector domains. Civil-society and academic participants mainly critiqued the biomedical and technical funding focus of philanthropic organisations; the characterisation of philanthropic funding as a self-sustaining industry came primarily from stakeholders from the government sector. Nonetheless, across actor domains, philanthropic influence was widely described as simultaneously enabling and constraining, providing resources and continuity while shaping the criteria of what counts as a legitimate intervention and concentrating agenda-setting power in the hands of private, unelected actors.

Much of the existing literature on philanthropic influence in global health has focused on low- and middle-income countries as primary sites of intervention, where international philanthropic foundations are typically analysed as external actors shaping policies, priorities and governance arrangements (18, 19, 39). Within this body of work, influence is commonly conceptualised along the so-called Global North–South lines, with private foundations positioned as powerful donors acting upon recipient contexts. This perspective has generated important insights into agenda-setting, priority shifts and governance effects in recipient countries, but has also implicitly framed philanthropic power as something that primarily operates outside of donor countries themselves.

The findings of this study challenge and complicate this framing. The donor/recipient binary that has structured much of the philanthropic influence literature is not merely descriptive convenience, but carries analytical consequences, implicitly locating philanthropic power “elsewhere” and treating donor-country contexts as sites of resource mobilisation and partnership rather than fields in which philanthropic influence itself operates. The present findings unsettle this assumption: participants described processes of strategic alignment, organisational dependency, thematic narrowing and governance fragmentation that closely mirror those identified in studies of philanthropic engagement in low- and middle-income contexts. A marked increase in the two foundations’ documented grant-making activity in Germany since the mid-2010s (32, 33) is consistent with participants’ accounts of a growing and increasingly structured institutional presence.

Rather than treating this as evidence that donor countries are simply ‘also affected’, the findings suggest that the donor/recipient distinction is itself an inadequate analytical lens—one that obscures how philanthropic power operates structurally and transnationally, across interconnected governance spaces, rather than unidirectionally from North to South. The empirical contribution of this study therefore lies in demonstrating that these dynamics are structurally embedded within the broader architecture of global health governance, and that understanding them fully requires moving beyond the donor/recipient frame. Germany does not merely function as a source of funding or political support for global health initiatives but emerges as a site in which philanthropic influence actively reshapes institutional relations, priorities and modes of coordination.

This interpretation resonates with broader critiques of philanthrocapitalism, which emphasise that the power of large foundations lies in their capacity to leverage financial resources to shape wider systems of governance, mobilise additional actors and influence policy environments beyond the immediate sites of intervention (40–42). In the German context, philanthropic engagement is closely embedded within domestic policy processes, institutional networks and funding landscapes, enabling forms of influence that are indirect, relational and often normalised within the field, making them less visible but no less consequential. By foregrounding these dynamics, the study contributes to an understanding of philanthropic power as a structural feature of global health governance that operates through the mobilisation and conversion of different forms of capital (40–42).

The concept of network diplomacy, introduced by de Bengy Puyvallée, Storeng and Rushton (21), describes how the Gates Foundation strategically embeds itself within governmental, institutional and societal networks in European donor countries and offers a framework to apply to the present findings. Participants described foundations as actors that structure networks, convene diverse stakeholders and shape patterns of interaction across the field through partnerships, indirect funding and convening—mechanisms that align closely with the network-based governance processes identified in that work. The German case thus appears not as an outlier but as part of a broader European pattern in which philanthropic actors operate through embedded, relational forms of governance.

This study extends the concept of network diplomacy by shifting the analytical perspective from institutional strategies to actor perceptions within the field. While de Bengy Puyvallée et al. (21) focus on how philanthropic organisations strategically cultivate relationships with donor governments and mobilise co-investment, the present findings highlight how these practices are interpreted, negotiated and, at times, critically reflected upon by actors within the domestic global health landscape. Network-based forms of influence are not experienced as neutral coordination mechanisms but are often associated with asymmetries in visibility, access and agenda-setting power. By funding research institutions, supporting advocacy organisations and engaging with policy networks, philanthropic actors contribute to shaping the environments within which policy debates take place, reinforcing the observation of de Bengy Puyvallée et al. (21) that such influence is cumulative and self-reinforcing: as certain priorities and approaches gain dominance, alternative perspectives become progressively marginalised.

A further finding concerns how participants interpreted the growing influence of international philanthropic foundations not primarily as a problem of private power, but as a consequence of structural weaknesses in public financing for global health. Several interviewees described their prominence as emerging from a vacuum created by declining or insufficient public investment in multilateral institutions. In this reading, philanthropic actors do not simply impose priorities onto the field but occupy spaces that public actors have partially vacated. This shifts the analytical lens in important ways. Much of the existing literature conceptualises philanthropic influence in terms of the expanding power of private actors and the risks this poses for democratic governance, accountability and equity (2, 13, 15). More broadly, scholarship on non-state actors in governance has highlighted that private actors can acquire forms of public authority through financial resources, expertise, networks, and participation in policy processes, while lacking the electoral mandates and conventional accountability mechanisms associated with state institutions (43, 44). This raises questions not only about the extent of philanthropic influence, but also about the basis of its legitimacy and the mechanisms through which such influence can be rendered transparent and accountable. From this perspective, the legitimacy of philanthropic involvement cannot be reduced to whether foundations exercise influence, but also concerns the conditions under which private authority is integrated into public governance and subjected to scrutiny.

While these concerns remain highly relevant, the findings in this study highlight that such influence cannot be fully understood without taking into account the broader political and economic conditions that enable it. Philanthropic dominance thus appears less as an autonomous driver of change and more as a relational phenomenon that is co-produced by the retrenchment, fragmentation or strategic repositioning of public actors. This interpretation resonates with critical perspectives on philanthrocapitalism, which emphasise that the growing role of private foundations is closely linked to wider transformations in the global political economy, including rising wealth concentration and shifting responsibilities between state and non-state actors (39, 45). This compensatory role is inherently ambivalent: philanthropic funding can stabilise programmes, sustain political attention and mobilise resources, while simultaneously reinforcing the very dynamics that make such interventions necessary, including dependency, fragmented governance and limited accountability.

The findings therefore point to a structural paradox. Philanthropic foundations are perceived as both stabilising and transforming the global health landscape: providing essential resources that enable continued engagement in times of fiscal constraint, while reshaping priorities, governance arrangements and actor relations in ways that may constrain pluralism and long-term systemic approaches. This dual role reflects the tension between short-term functionality and long-term structural implications that characterises contemporary global health governance (46). The prominence of philanthropic actors can thus be understood as indicative of a broader reconfiguration of state responsibilities and multilateral cooperation—one that demands ongoing attention to the conditions under which public actors rely on private resources to fulfil governance functions.

### Implications

The findings carry implications at multiple levels. For research, they underscore the value of examining philanthropic influence within donor-country contexts, where systematic actor-centred analysis remains limited. Comparative research across European donor countries would help clarify which of the patterns observed here are specific to the German context and which reflect broader structural tendencies, as suggested by the network diplomacy literature. For Germany policy makers, the findings point to the importance of developing clearer frameworks governing the terms and scope of philanthropic engagement in national policy contexts, particularly as Berlin consolidates its role as a hub for transnational health diplomacy and the institutional presence of international philanthropic foundations in Germany may continue to grow. More broadly, the dynamics identified in this study may resonate, to varying degrees, in other high-income donor countries where international philanthropic organisations play an active and structurally embedded role. Comparative research across contexts where foundations such as the Gates Foundation and Wellcome Trust have been embedded for considerably longer, such as the United States and the United Kingdom, as well as contexts where philanthropic presence is more nascent, would help clarify which dynamics are specific to the German context and which reflected broader structural tendencies. For practice, the findings draw attention to the governance implications of growing dependency on private foundations at a moment of contracting public budgets. The thematic concentration associated with current philanthropic priorities and the perceived marginalisation of structural health approaches, rights-based frameworks and health system strengthening points to the importance of deliberate efforts to preserve pluralism in agenda-setting. This includes greater transparency regarding the terms and scope of philanthropic partnerships and governance arrangements that create meaningful space for actors and perspectives that fall outside dominant philanthropic funding logics.

### Limitations and future research

Several limitations of this study warrant mention. The sample of 11 participants, while purposefully constructed to ensure representation across five actor domains, reflects the exploratory scope of the study and the constraints of a Master’s thesis project. Recruitment outcomes varied across actor domains as described in the Methods section. The small sample size and number of participants across actor domains may have limited the diversity of perspectives captured and resulted in some stakeholder perspectives being less represented than others. The findings should therefore be interpreted as reflecting the perspectives of the recruited participants rather than the full range of views present across Germany’s global health landscape. Snowball sampling, appropriate for accessing a specialised expert community, may have introduced a bias toward participants with existing proximity to philanthropic networks. The study relies exclusively on interview data, capturing perceptions rather than documented funding flows or governance outcomes; combining qualitative approaches with document analysis or network mapping could deepen these findings. The sample size did not permit analysis of variation in perceptions by participant characteristics such as sex or gender, age, or seniority—factors that may shape how philanthropic influence is experienced and that warrant attention in future research. Finally, the interviews were conducted at a specific moment, and perceptions may have evolved given the rapidly shifting geopolitical and fiscal context—including ODA contractions, the US withdrawal from the WHO, and the Gates Foundation’s announced closure in 2045—developments that lend additional timeliness to the questions this study raises.

## Conclusion

This study offers an actor-centred analysis of how international philanthropic foundations are perceived within Germany’s domestic global health landscape—a donor-country context that has received comparatively little systematic attention in the philanthropic influence literature. The findings reveal a structurally ambivalent picture: foundations are simultaneously valued as enablers of continuity, expertise and political momentum, and critiqued as concentrators of agenda-setting power operating outside democratic accountability. Critically, the dynamics identified here—strategic alignment, thematic narrowing, governance fragmentation and organisational dependency—are not unique to low- and middle-income country contexts but are structurally embedded within the broader architecture of global health governance, transcending the donor/recipient binary that often frames this field of inquiry.

As public financing for global health contracts and philanthropic actors assume an increasingly prominent role in filling resulting gaps, the questions raised by this study become more rather than less urgent. Understanding how philanthropic influence operates within donor countries by shaping priorities, actor relations and the boundaries of legitimate intervention, is essential for developing governance frameworks that preserve pluralism, accountability and equity in global health. The German case offers one window into these dynamics; comparative research across donor contexts will be necessary to fully illuminate their scope and consequences.

## Data Availability

The raw data supporting the conclusions of this article will be made available by the authors, without undue reservation.
