# Supplementary material for: Private capital in a public field: a qualitative study of philanthropic influence in Germany’s global health governance
Source: Front Public Health. 2026 Sep 21;14:1960171. doi: 10.3389/fpubh.2026.1960171 (PMC13635097; doi:10.3389/fpubh.2026.1960171)
Supplement: Supplementary file 1 [file Data_Sheet_1.PDF]

International Philanthropy's Footprint in Germany's Global Health Landscape:

A Qualitative Multi-Actor Study Covering the Last Fifteen Years

Interview guide

Introduction

Thank you very much for agreeing to take part in this interview, I really appreciate your time.

As you know, the aim of my research is to explore how international philanthropic organisations, in particular the Gates Foundation and the Wellcome Trust, are perceived by different actors within Germany's global health landscape. I'm particularly interested in your perspective on how these organisations have engaged with the field over the past 15 years, and the nature of their role and influence from your position.

Just to briefly confirm, you've provided written consent to take part in this interview and for the conversation to be audio recorded. Are you still comfortable with this?

The interview will take around 45 to 60 minutes. Please feel free to skip any questions you do not wish to answer, and you can stop the interview at any time without giving a reason. If you would prefer to express something in German during the interview, please feel free to do so. I will translate your statements into English word for word in the transcript later.

In terms of the structure of the interview, we'll begin with a few general questions so I can better understand your role and experience in the global health field, particularly in the German context. From there, we'll explore how you've observed the evolving presence and influence of international philanthropic organisations, in particular the Gates Foundation and the Wellcome Trust over the past 15 years. The interview is structured around different dimensions of influence, such as expertise, networks, visibility, and funding. Toward the end, I'll ask a reflective question as well as your thoughts on how these dynamics may develop in the future.

Before I begin the recording, do you have any questions about the interview process or this research project?

[…]
Start audio recording and proceed with the interview.

Note for me:

- Questions or prompts in purple are intended exclusively for representatives of the Gates Foundation and the Wellcome Trust.
- Questions or prompts in yellow are directed to all participants, excluding representatives of the Gates Foundation and the Wellcome Trust.

Questions
1. ***What are participants' perspectives on Germany's global health landscape, and how do they position themselves and their organisations within this field?***<br><br>Note: This section aims to explore participants' general understanding of Germany's global health engagement, their own role and institutional context, and any existing links to philanthropic actors. It avoids foregrounding the influence of foundations too strongly at this stage, to allow participants to raise relevant connections or experiences naturally. Questions are framed broadly to capture institutional positioning, networks, and types of collaboration in the field.
1.1 To begin with, could you briefly describe your role and area of work in the context of the German global health landscape?<br><br>• Introduce the organisation and its focus in the field of global health in Germany<br><br>1.2.1 How does your organisation interact or collaborate with actors from Germany in the field of global health?<br><br>1.2.2 Could you describe whether and in what way you have already had contact with international philanthropic organisations in your role?<br><br>1.3 How would you describe the current global health landscape in Germany?<br>• Prompts:<br>○ key priorities, debates, or tensions<br>○ actors or institutions do you see as particularly active or influential<br>○ level of coordination or fragmentation across actors
1. How do global-health actors, perceive the evolving presence and role of international philanthropic organisations in the German global health landscape over the past 15 years?<br><br>Note: This first part of the interview focuses on general changes in the German global health landscape brought about by the presence and role of the Gates Foundation and the Wellcome Trust. The aim is to introduce the interviewees to the main topic. The discussion is then opened to gather initial impressions of possible forms of influence based on Bourdieu's forms of capital (economic, cultural, social, symbolic). This section prepares the ground for an in-depth analysis of the individual dimensions in the rest of the interview.
1.1 How has the role and presence of international philanthropic organisations in the German global health context changed over the last 15 years?<br><br>Two of the biggest the biggest international philanthropic organisations, who have also been most active in Germany in recent years, are the Gates Foundation and the Wellcome Trust. The next set of questions therefore focus specifically on these two organisations. However, if at any point you

want to add something about international philanthropic organisations more generally in Germany, please do so.

1.2 How has the role and presence of the Gates Foundation and the Wellcome Trust in the German global health context changed over the last 15 years?

- If the participants speaks about one of the organisations, subsequently prompt them about the other organisation.
- The Gates Foundation established an office in Berlin in 2018 / The Wellcome Trust established an office in Berlin in 2019. Could you please briefly explain the goal and strategic thinking about this move?
- In this context, do you see any differences and/or similarities between the Gates Foundation and the Wellcome Trust? If yes, can you give me an example please?

1.3 In which settings or processes have the Gates Foundation and/or the Wellcome Trust become particularly visible in Germany's global health field over the past 15 years (symbolic capital)

- How has the presence of the Gates Foundation and the Wellcome Trust influenced the focus areas of global health in Germany)?
- If participants ask for clarification or an example: e.g. a shift from health systems or equity to innovation or technical solutions.
- How has the Gates Foundation or the Wellcome Trust's involvement in Germany changed, particularly regarding a possible shift from a rather reserved role to a visible, institutional presence (e.g. by opening an office)?

1.4 Over the last 15 years, which networks and institutions have the Gates Foundation and Wellcome Trust sought to access and establish themselves within? (Social capital)

- Would you say that Gates Foundation and Welcome Trust work together or alongside each other in this process?
- After establishing your locations in Germany, who did you seek contact and cooperation with?
- How has the structure of networks (e.g. policy forums, research collaborations) changed because of the presence of Gates Foundation and Wellcome Trust in the last 15 years?

1.5 What kind of knowledge or expertise have the Gates Foundation and Wellcome Trust brought into the German global health field over the last 15 years?

- E.g. technical, strategic, or scientific-methodological expertise?
- How is the expertise perceived, in terms of its unique value or influence, for example compared to national actors (e.g. RKI)?

1.6 How have the Gates Foundation and the Wellcome Trust positioned themselves financially in Germany's global health field over the past 15 years? (economic capital)

- What are the overarching goals or intentions that shape decisions about how your organization directs financial support? For example, in terms of strengthening existing structures, priority setting, and so on.?
- In your perspective, what are the main pros or benefits, and main negatives or challenges, about the financial contributions from the Gates Foundation and Wellcome Trust, particularly in terms of setting priorities??

2. *How do global-health actors view the implications of this shift in international philanthropic organisations' presence in Germany for the roles and responsibilities of other stakeholders in Germany's global-health landscape?*

Note: This section examines how the growing presence of the Gates Foundation and the Wellcome Trust has changed the roles and responsibilities of other actors in the German global health field. The questions aim to show how social and cultural capital intervene in institutional roles and how this is reshaping the structure of the field from the perspective of the actors.

Thank you very much for your insights so far. We briefly discussed the integration of Gates and Wellcome into network structures in the global health landscape in Germany and the expertise they bring to the table. I would like to delve a little deeper into these two topics in the following questions.

3.1.1 In what ways has the integration of the Gates Foundation and Wellcome Trust into German networks over the last 15 years influenced or changed the roles or visibility of existing actors?

- Could you provide an example for this?
- In that example what were indicators for a change in influence?

3.1.2 How do you choose which actors to work with in Germany?

- What are the key considerations or criteria used to identify key partners, e.g. political relevance, scientific reputation, network position?
- Have your key partners in Germany changed over the last 15 years, and if so, what drove that shift?

3.2 Have partnerships or initiatives with the Gates Foundation or the Wellcome Trust in the German global health field over the past 15 years influenced the nature of institutional alliances and alignments in this context?

- Have there been any cases where this has contributed to greater convergence or divergence or division, in terms of content or institutional alignment? If so, could you please give an example?
- Were there any coalitions or partnerships that would not have come about without Gates Foundation and or Wellcome Trust, if so, could you name an example?

3.3 How have the Gates Foundation and the Wellcome Trust influenced strategic content and responsibilities in the German global health sector over the past 15 years?

- Some actors shape strategic content and responsibilities proactively by setting agendas ("control-oriented"), while others rather respond to existing developments ("cooperation-oriented").
  How would you assess the role of the Gates Foundation and the Wellcome Trust in this regard in the German global health sector?
- To what extent have German actors adapted their own priorities or approaches in response to the agendas or preferences of the Gates Foundation or the Wellcome Trust??

- ***4. How do global-health actors perceive changes in the distribution of influence, priority-setting, and resource allocation in the context of the evolving role of international philanthropic organizations in Germany's global-health landscape?***

Note: This section focuses on perceived changes in the distribution of influence, thematic priorities and financial resources in the German global health field due to the increasing presence of international foundations. The focus is on how symbolic capital and economic capital influence the distribution of roles and power relations between actors.

We have come to the last section of my interview. In this part, I would like to return to the topic of funding and legitimacy in connection with Gates Foundation and Wellcome Trust.

4.1 How has funding from the Gates Foundation and the Wellcome Trust influenced the focus in the German global health sector changed over the past 15 years? For example, are there any topics that have received more or less attention?

4.2 To what extent have the financial networks or partnerships supported by the Gates Foundation or the Wellcome Trust, facilitated the visibility or integration of certain actors in the German global health sector, who may have otherwise faced challenges in establishing themselves?

- Can you think of examples where new actors that gained access to policy spaces, funding rounds, or visibility through such connections with by the Gates Foundation or the Wellcome Trust?
- To what extent have stakeholders changed their positioning to attract the interest of the Gates Foundation or the Wellcome Trust?
-

4.3 As a concluding question for this section, how is the role of Gates Foundation and Wellcome Trust in the German global health field legitimised, and by whom?

- Prompt if needed: funding; innovation; global networks; expertise; partnerships; support by political leaders; support by academia; media framing; public acceptance, etc
- As you likely know, **legitimacy** and **accountability** are closely related. To what extent, in your perspective, are there mechanisms in place to ensure accountability by these organisations to the public or other stakeholders?

(If examples/clarification needed: e.g. mechanisms for them to explain, justify, and take responsibility for decisions and action.)
5. Conclusion and wrap up
Thank you very much for agreeing to be interviewed for my master's thesis. Your insights and contributions to my research are very interesting.<br><br>5.1 To wrap up, considering the recent change in government, would you like to share any expectations or thoughts on how the collaboration between German stakeholders in the global health landscape and international philanthropic organisations like the Gates Foundation and Wellcome Trust might develop over the next five years?<br><br>5.2 We are now at the end of the interview. Is there anything else you would like to add, any thoughts, concerns, or issues we haven't touched on?
Thank you again for your time and valuable input. I'd be happy to keep you informed about the progress and outcomes of the project, especially in case of publication.<br><br>I am going to transcribe these interviews. Would you like to take the opportunity to review your transcript?<br><br>Also, I am wondering if you would have anyone else that you think I should interview? If so could you please provide me with their name and contact information?
